# Editorial: Non-neuronal cell heterogeneity in the nervous system during health and disease

**DOI:** 10.3389/fncel.2022.1047296

**Published:** 2022-10-19

**Authors:** Greg J. Duncan, Peggy Assinck, Jo Anne Stratton, Bahareh Ajami, Jason R. Plemel

**Affiliations:** ^1^Jungers Center for Neurosciences Research, Department of Neurology, Oregon Health and Science University, Portland, OR, United States; ^2^Wellcome Trust - MRC Cambridge Stem Cell Institute, University of Cambridge, Cambridge, United Kingdom; ^3^Neuroimmunology Group, Montreal Neurological Institute and Department of Neurology and Neurosurgery, McGill University, Montreal, QC, Canada; ^4^Department of Molecular Microbiology and Immunology (MMI) and Behavioral and Systems Neuroscience, Oregon Health and Science University, Portland, OR, United States; ^5^Department of Medicine, Division of Neurology, University of Alberta, Edmonton, AB, Canada; ^6^Neuroscience and Mental Health Institute, University of Alberta, Edmonton, AB, Canada; ^7^Department of Medical Microbiology and Immunology, University of Alberta, Edmonton, AB, Canada

**Keywords:** heterogeneity, glia, scRNAseq, astrocytes, oligodendroglia, microglia, ependymal cells, meninges

Non-neuronal cells, which are just as numerous as neurons within central nervous system (CNS), have been historically understudied (Barres, 2008; Allen and Lyons, 2018). This is largely attributable to the precision by which electrophysiologists could measure neuronal activity. It was these measurements that first revealed the incredible diversity of neurons in their firing patterns, stimuli, connectivity and ultimately function, making neurons the center of neuroscience research. In recent years, non-neuronal cells have received increasing attention and considerable diversity has also been revealed by new single-cell approaches, most notably single-cell (sc) or single-nucleus (sn) RNA sequencing (RNAseq). The work contained within this Research Topic discusses diversity within major non-neuronal cell types in the CNS: astrocytes, oligodendroglia, ependymal cells and microglia. It also touches on the diversity of the meninges, which span both the CNS (leptomeninges) and the periphery (dura). These publications highlight the importance of non-neuronal diversity in the development, function, and disease progression within the nervous system.

How the brain is wired in development and damaged in disease are fundamental questions to treat those suffering from neurological conditions. Neuronal wiring is established during early embryonic development when neuronal progenitors migrate from the neuroepithelium to precise locations, adopt specific cell fates, and form circuits. The findings that non-neuronal cells like astrocytes exhibit regional and layer-specific heterogeneity (Batiuk et al., 2020; Bayraktar et al., 2020), considerable diversity in response to neuro-inflammation (Hasel et al., 2021) and disease (Itoh et al., 2018; Diaz-Castro et al., 2019) has raised great interest in how these cells may be altering neuronal circuits. In a comprehensive review on inflammatory astrocytes, Reed and Kuipers describe the bidirectional feedback between astrocytes and inflammatory cells which can trigger neurodegeneration and also postulate that these inflammatory states could be targeted to reduce neurodegeneration. Moulson et al. reviewed astrocyte diversity in the context of CNS pathologies such as ischemic stroke, CNS demyelination, and traumatic injury and introduced the idea that the observed diversity might represent plasticity in these pathologies. Lastly, Lo et al. highlight the advancement of single-cell approaches and their challenges for characterizing astrocyte heterogeneity. They conclude that astrocytes display heterogeneity in health but with indistinct cellular hierarchies relative to neurons and are polarized to a number of different subpopulations following demyelination, not merely neuroprotective or neurodegenerative.

Microglia are highly plastic cells in which even subtle changes in homeostasis alter gene expression, morphology and function. Huarte et al. review microglia heterogeneity and highlight how single-cell technologies have allowed for the identification and further stratification of novel subsets of microglia in health and disease. Many of these microglial states are context-derived, and thus may reflect changes to their niche. Oligodendrocyte progenitor cell (OPC) diversity may also arise from changes to their niche and not necessarily from intrinsic differences between cells. In a mini review, Sherafet et al. highlight that diversity in OPCs are typically a process of age, differentiation state and location and argue that OPCs have not been shown to be segregated into functionally distinct subtypes. Sherafat et al. propose a number of factors in the pericellular environment influence OPC diversity and highlight the proximity to the vasculature to be critical for OPC migration, differentiation and vulnerability to demyelinated disease. OPCs and microglia are both shaped by their microenvironment and are poised to respond precisely and rapidly to changes in homeostasis.

Single-cell technologies have revealed an underappreciated diversity in non-neuronal cells, but also provided insights into novel functions of cellular subpopulations. MacDonald et al. use available data in single cell transcriptomic datasets to propose a critical role of ependymal cells in metal ion homeostasis. Additionally, they identify transcriptionally distinct subtypes of ependymal cells dependent on spatial location in the CNS. Like ependymal cells, the meninges are often an overlooked population that were long thought to be primarily for structural support and physical protection. Derk et al. thoroughly review the cellular composition of the meninges, their developmental origins, and functions. During health, the meninges regulates the entry and exit of solutes from the brain, and in disease or injury regulate scarring and inflammation (Dorrier et al., 2021). Bouadi and Tay argue single-cell approaches will be key to understanding how to reduce neuro-inflammation and scarring that accumulates around multi-electrode arrays and neuroprosthetics. Scarring diminishes the effectiveness of these structures over time. They postulate that these devices offer enormous therapeutic promise in pathologies like hearing loss or spinal cord repair.

The articles within this special edition highlight the considerable progress that has been made on identifying non-neuronal diversity, aided in large part by scRNAseq. Additionally, novel technologies will continue to expand the types of data that can be derived to distinguish diverse cell populations. For example, spatial transcriptomics has now been used to identify astrocyte heterogeneity over cortical layers (Bayraktar et al., 2020) and will likely be applied to other non-neuronal cell types. RABID-seq offers an intriguing approach to analyze cell-to-cell interactions (Clark et al., 2021). However, as valuable as these approaches are, mRNA levels often have a low correlation to subsequent protein expression (Tian et al., 2004; Koussounadis et al., 2015). Single-cell proteomics on large numbers of cells may become feasible as advances in mass spectroscopy and barcoding continue (Perkel, 2021). It will also be crucial in coming years to move beyond identifying transcriptionally diverse populations and to truly define their functional roles. To do so, targeted genetic access for specific cell subpopulations will be needed. scRNAseq often identifies unique genes which are expressed in particular subpopulations that could be used to develop Cre-expressing lines (Tran et al., 2019). It is an exciting time to study non-neuronal cells and novel technologies are providing insights into the diversity and critical functions of these cells.

Articles from within this editorial have articulated the importance of carefully choosing appropriate terminology to describe diversity between and within cell populations (Figure 1). Terms such as heterogeneity, cell state, diversity, plasticity, maturation and others may be overused without clear definitions. Defining these terms through consensus with carefully chosen terminology will lead to precise biological conclusions that are imperative to deepen our understanding of cell heterogeneity and function.

**Figure 1:**
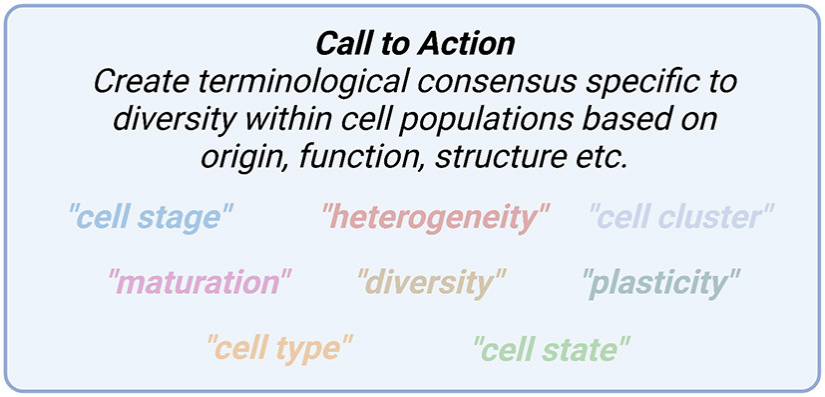
A number of terms to describe cell state differ between researchers and fields and require more precise definitions.

## Author contributions

GD, PA, and JS planned the outline for the Editorial. GD composed the initial draft of the Editorial. PA drafted the figure. PA, JS, BA, and JP all edited and improved the Editorial. All authors approved the final manuscript.

## Funding

GD is funded by a National Multiple Sclerosis Career Transition Award (TA-2105-37636). PA is supported by a Banting Postdoctoral Canadian Institute for Health Research (CIHR) Award. BA is supported by Michael J Fox Foundation (MJFF) and Alzheimer's Association Research Grants. JS is funded by an FRQS Junior 1 Award. JP is supported by a Canada Research Chair in Glial Neuroimmunology.

## Conflict of interest

The authors declare that the research was conducted in the absence of any commercial or financial relationships that could be construed as a potential conflict of interest.

## Publisher's note

All claims expressed in this article are solely those of the authors and do not necessarily represent those of their affiliated organizations, or those of the publisher, the editors and the reviewers. Any product that may be evaluated in this article, or claim that may be made by its manufacturer, is not guaranteed or endorsed by the publisher.
